# Hypoglycemic Activity of Medicinal Plants Used among the Cakchiquels in Guatemala for the Treatment of Type 2 Diabetes

**DOI:** 10.1155/2019/2168603

**Published:** 2019-01-01

**Authors:** Adolfo Andrade-Cetto, Elda Carola Cruz, Christian Alan Cabello-Hernández, René Cárdenas-Vázquez

**Affiliations:** ^1^Laboratorio de Etnofarmacología, Facultad de Ciencias, Universidad Nacional Autónoma de México, Avenida Universidad 3000, 4510, Ciudad de México, Mexico; ^2^Laboratorio de Biología Animal Experimental, Universidad Nacional Autónoma de México, 04510, Ciudad de México, Mexico

## Abstract

Type 2 diabetes (T2D) is a major health problem worldwide. In this condition, the organism can produce insulin but becomes resistant to it; thus the insulin is ineffective. High blood glucose levels are a result of insulin resistance and insulin deficiency; they produce diabetes-associated complications such as kidney failure, blindness, cardiovascular disease, and lower-limb amputation. In Guatemala, there were over 752.700 cases of the disease in 2017 with prevalence of 8.4 (IDF, 2017). The use of plants for medicinal purposes has been practiced in the country since pre-Hispanic times. Among the Cakchiquels, the aerial parts of* Hamelia patens* Jacq.,* Neurolaena lobata* (L.) R.Br. ex Cass., and* Solanum americanum* Mill. and the cortex of* Croton guatemalensis* Lotsy are used to treat type 2 diabetes. The aim of the present study was to confirm the hypoglycemic effect of the plants under normal conditions and under maltose and sucrose tolerance tests, as well as to test the activity of the plant extracts in vitro against alpha-glucosidases types I and II. In agreement with the traditional usage of the plants, in normal conditions without a sugar load, the extracts produced a statistically significant hypoglycemic effect similar to the control drug glibenclamide. When the sugar load was maltose, only* Croton* and* Solanum* produced a statistically significant (*p* < 0.05) hypoglycemic effect compared to the control drug, but when the sugar was sucrose,* Croton* and* Hamelia* produced a statistically significant effect (*p* < 0.05) beginning at 30 min compared to the control group, while* Solanum* did so at 60 min and* Neurolaena* at 90 min. In vitro assays showed that the extracts inhibited yeast* alpha-glucosidases* but not the rat intestinal ones. Of the tested plants,* Croton* exert an effect both under sugars' tests and under a normal tolerance test; these results suggest the potential use of this plant. The results presented here provided evidence based on the use of these plants as hypoglycemic agents in the treatment of type 2 diabetes.

## 1. Introduction

Diabetes is a chronic condition that occurs when the body cannot produce sufficient insulin or use it appropriately. In this condition, the organism can produce insulin but becomes resistant to it, causing the insulin to be ineffective. High blood glucose levels are a result of insulin resistance and insulin deficiency. In T2D the insulin levels may become insufficient; thus high blood glucose levels are a consequence of insulin resistance and insulin deficiency. Type 2 diabetics suffer from insulin resistance and usually relative rather than absolute insulin deficiency. These individuals may not require insulin treatment to survive initially and often throughout their lifetimes [1, 2].

In 2017, the International Diabetes Federation (IDF) estimated that nearly half a billion people were living with diabetes worldwide. Low- and middle-income countries carry almost 80% of the burden of diabetes and diabetes-associated complications, including cardiovascular disease, blindness, kidney failure, and lower-limb amputation, which are major causes of disability, a low quality of life, and premature death [3].

In diabetic patients, postprandial hyperglycemia initiates a cascade of proatherogenic and prothrombotic events, which are independent risk factors for cardiovascular disease, stroke, and mortality. A fast rise in the glucose levels has been shown to increase low-grade inflammation. Postprandial hyperglycemia may produce harmful effects on *β*-cells (glucotoxicity) and has been shown to deteriorate insulin sensitivity in the muscular cells [4]. Alpha-glucosidase inhibitors (AGIs) lower postprandial blood glucose concentrations; they act as competitive inhibitors with high affinity for alpha-glucosidases. AGIs must be present at the site of enzymatic action at the same time as the carbohydrates; this action slows glucose input into systemic circulation. Acarbose, a therapeutically used AGI, has efficacy against various alpha-glucosidases, primarily glucoamylase, followed by sucrase, maltase, and dextranase. It also inhibits alpha-amylase but has no effect on beta-glucosidases, such as lactase [4].

In Guatemala, more than 752,700 cases of the disease were reported in 2017, with prevalence of 8.4 [1]. The use of medicinal plants has been practiced in Guatemala since pre-Hispanic times. The low incomes of the indigenous populations are associated with the prevalence of type 2 diabetes, which accounts for 33% of the mortality in the country. These factors make diabetic people use medicinal plants to treat this illness [5].

In a previous ethnopharmacological field study, we documented the use of nearly 50 plants to treat type 2 diabetes among the Cakchiquels. For that propose, we interviewed 128 diabetic people and confirmed that 91% of them used medicinal plants to treat the illness. After qualitative and quantitative (disease consensus index) analysis of the interviews and the data, the plants* Croton guatemalensis* Lotsy,* Hamelia patens* Jacq.,* Neurolaena lobata* (L.) R.Br. ex Cass., and* Solanum americanum* Mill. were recommended for further investigation of their hypoglycemic effects [6].

The aim of the present study is to confirm the hypoglycemic effects of the plants under normal, maltose, and sucrose tolerance test conditions and to test the activity of the extracts in vitro against alpha-glucosidase types I and II.

*Brief Descriptions of the Studied Plants*.* Croton guatemalensis* Lotsy (Cg) is a small tree up to 6 m high distributed in the tropical and subtropical areas of the Americas, including Mexico, Colombia, Ecuador, and Guatemala. The plant is reported for its antinociceptive effect [7] and its antiplasmodial and cytotoxic effects [8]; there are no reports in the international literature on its hypoglycemic effect.* Hamelia patens* Jacq (Hp) is a shrub up to 3 m high that can be found along rivers in warm and semiwarm climates between 8 and 1100 meters above sea level. This plant is associated with secondary vegetation of oak and deciduous forests. It inhabits areas from the southern parts of Florida to Mexico, Central America, and South America in Bolivia. An acute hypoglycemic effect of the plant collected in Mexico has been reported by [9]. The inhibition of type 1 alpha-glucosidases and the isolation of (6E,10E,14E,18E)-2,6,10,14,18,23-hexamethyl-2,6,10,14,18,22-tetracosahexaene, *β*-sitosterol, and stigmasterol were done by [10]. The antihyperglycemic effect of the plant associated with the compounds epicatechin and chlorogenic acid was also demonstrated by [11].

*Solanum americanum* Mill. (Sa) is a herbaceous plant, erect or creeping up to 1.5 meters high, with a branched stem with bent hairs, and inhabits low deciduous forests, high evergreen forests, coastal dunes, and sometimes xerophilous scrub. The phytochemical composition and its action on type 1 glucosidases have been reported [12]; the inflammatory response of four extracts of ripe fruits was demonstrated by [13]; the plant was also reported as an anthelmintic remedy in Gabon [14].

*Neurolaena lobata* (L.) R.Br. (Nl) is a herb or bush which is erect and stems up to 3 m high and inhabits warm and semiwarm climates between 5 and 1200 m.a.s.l. This plant is distributed from Mexico to the northern part of South America; no works in the international literature have investigated its hypoglycemic activity; the plant is reported as an alternative therapy for gastric hypersecretion [15], for its antimalarial activity [16], and as an anti-inflammatory [17].

## 2. Materials and Methods

### 2.1. Plant Collection and Extract Preparation

Based on previous ethnobotanical studies [6], the four plants were gathered from the Department of Chimaltenango, which is located 56 km west of Guatemala City at 1,817 m.a.s.l. at 14°39'38” N and 90°49'10” W. With the help of diabetic patients and specialists, the plants were collected from the following towns: Santa Apolonia, San Martín Jilotepeque, San José Poaquil, and San Miguel Pochuta. The plant identities were determined by David Mendieta and Max Mérida of San Carlos University in Guatemala. Vouchers were deposited at the Deshidrafarmy-Farmaya Herbarium:* Hamelia patens*, CFEH 1242;* Neurolaena lobate*, CFEH 1239;* Croton guatemalensis*, CFEH 1259; and* Solanum americanum*, CFEH 1262.

Different extracts were prepared to study the plants' hypoglycemic effect. A water extract similar to the traditionally used tea (W) was prepared by boiling 20 g of dry plant material with 500 ml of water, followed by filtration and lyophilization, as previously described [18, 19]. An ethanol-water extract (EW) was prepared by adding 20 g of the plant material to 500 ml of an ethanol and water mixture (50:50); then, the mixture was heated at 40°C for four hours and filtered three times, followed by evaporation in a Buchi rotary evaporator. All extracts were stored at -40°C prior to use.

Based on field calculations [6], the final yield of each extract was considered as the traditional dose used by one person of a weight of 70 kg; the equivalent of the traditional used doses was administered to the animals.

### 2.2. Experimental Animals

All methods used in this study were approved by the Internal Council of the Facultad de Ciencias, Universidad Nacional Autónoma de México. Animals were handled according to the procedures outlined by the Committee for the Update of the Guide for the Care and Use of Laboratory Animals (2015).

Wistar rats weighing 200-250 g were obtained from the Bioterium of the Facultad de Ciencias, UNAM, and were acclimated with free access to food and water for at least one week in an air-conditioned room (25°C with 55% humidity) on a 12 h light-dark cycle prior to the experiments.

Experimental diabetes was induced as previously described [20]. Briefly, the rats were made to fast overnight and injected intravenously with 65 mg/kg of streptozotocin (STZ) in a citrate buffer (Sigma, S0130), 15 min before they were injected intraperitoneally with 150 mg/kg of nicotinamide (NA) (Sigma, N3376). After 48 h, diabetes was identified by polydipsia, polyuria, and measuring nonfasting plasma glucose levels. Animals that did not develop glucose levels greater than 250 mg/dl were rejected.

### 2.3. Experiment 1: Acute Hypoglycemic Effect

As previously reported [18], hyperglycemic animals were divided into eleven groups (1-11) of six rats each. Group 1, the normal control group, orally received 1.5 ml of a physiological NaCl solution (vehicle), group 2, the hyperglycemic control, also received 1.5 ml of a physiological NaCl solution, and group 3, the positive control, received a standard oral hypoglycemic agent (glibenclamide, 5 mg/kg body weight (bw)) in the same vehicle. Groups 4 and 5 were given Cg (W 20 mg/kg and EW 30 mg/kg, respectively), groups 6 and 7 received Hp (W 36 mg/kg and EW 35 mg/kg, respectively), groups 8 and 9 received Nl (W 40 mg/kg and EW 57 mg/kg, respectively), and groups 10 and 11 received Sa (W 62 mg/kg and EW 77 mg/kg, respectively); all of these treatments were dissolved in 1.5 ml of a physiological NaCl solution.

### 2.4. Experiments: 2 and 3: Maltose and Sucrose Tolerance Tests.

For experiment 2, the hyperglycemic animals were classified into 7 groups (1-7) with six rats each [21]. All groups received a 3 g/kg maltose (a disaccharide formed from two units of glucose) solution 5 min after administration of the control drug or the extracts. Group 1, the normal control, received 1.5 ml of a physiological NaCl solution (vehicle), group 2, the hyperglycemic control, received 1.5 ml of a physiological NaCl solution, group 3, the positive control, was given the standard oral hypoglycemic agent acarbose (3 mg/kg), group 4 received Cg (W 20 mg/kg), group 5 received Hp (W 36 mg/kg), group 6 received Nl (W 40 mg/kg), and group 7 received Sa (W 62 mg/kg); all treatments were dissolved in 1.5 ml of a physiological NaCl solution. For experiment 3 [22], the same groups were formed, but the rats received a 2 mg/kg sucrose (a disaccharide composed of glucose and fructose) solution instead of maltose.

Blood samples were obtained from the tail vein. Glucose monitoring was performed and analyzed with glucose test strips and a glucometer (Accutrend® Plus) in duplicate. All groups were fed Purina Rodent Laboratory Chow 5001. Because all of the extracts exhibited hypoglycemic activity in experiment 1, the same doses of the W extract (similar to the traditionally used infusion) were selected for experiments 2 and 3.

### 2.5. Crude Small Intestine Extract

A crude extract of the rat intestine was prepared according to a previously described method [23] with modifications. The small intestines from 6 Wistar rats were dissected and washed twice with an ice-cold physiological saline solution (9% NaCl) and once with a 0.1 M potassium phosphate buffer (pH 7) with 5 mM EDTA. The mucosa from the washed small intestine was scraped and homogenized in a potassium phosphate buffer and then centrifuged at 21,000 x g for one hour. The precipitate was resuspended and incubated for 30 min in a 0.1 M potassium phosphate buffer (pH 7) containing 1% Triton X-100 and then centrifuged at 100,000 x g for 90 min. The supernatant was dialyzed against a 0.01 M potassium phosphate buffer (pH 7.0) for 24 h. The dialysate was lyophilized and stored at -20°C until needed.

### 2.6. In Vitro Glucosidase Assay

The enzyme activity was measured following a previously described procedure [24] with slight modifications. The activity was quantified by the amount of p-nitrophenol released from p-nitrophenyl-alpha-D-glucopyranoside. The assay contained a 0.1 M sodium phosphate buffer (pH 6.8) with 2 mM of p-4-nitrophenol glucopyranoside (p-NPGP) and 0.1 U of* alpha-glucosidase* from the crude extract* Saccharomyces cerevisiae* (Sigma® G5003-1KU) or the small intestine and the control drug (acarbose, Bayer) or experimental extracts at concentrations ranging from 0.2 *μ*g/ml to 20,000 *μ*g/ml in a 1 ml volume. The reaction was tracked for 480 sec; lectures were acquired every 15 sec at the 405 nm wavelength with a Beckman Coulter spectrophotometer (model DU-640).

### 2.7. Statistical Methods

The data were analyzed using an ANOVA test, followed by Fisher's post hoc test using the software Statistician. The plasma glucose levels were expressed as the mean (SEM); significance was considered at least with* p* < 0.05.

## 3. Results

### 3.1. Plant Yields

All extracts were prepared using 20 g of plant material in agreement with the Cakchiquels' traditional method. These people boil a fistful of the plant in 500 ml of water; as a result of direct measurements in the field (50 diabetic users), we determined that the mean for a fist was 20 g. The yields obtained starting with 20 g of plants were as follows:* Croton guatemalensis*: W = 1.4 g, EW = 2.1 g;* Hamelia patens*: W = 2.5 g, EW = 2.4 g;* Neurolaena lobata*: W = 2.8 g, EW = 3.9 g; and* Solanum americanum*: W = 4.3 g, EW = 5.3 g.

### 3.2. Experiment 1: Acute Hypoglycemic Effect

The results presented in Table 1 show that all tested extracts exert a hypoglycemic effect. When the blood glucose levels of the normal control group were compared with the levels of the hyperglycemic control, the hyperglycemic group had sustained elevated glucose levels that were stable through 180 min. The control drug glibenclamide exerted a sustained statistically significant hypoglycemic effect beginning at 60 min and continuing to 180 min, lowering the glucose 56% after 90 min. All the tested extracts presented a statistically significant (*p* < 0.05) hypoglycemic effect similar to glibenclamide since 30 min except for* Neurolaena lobata* EW, which produce a significant effect at 60 min. When the glucose values are analyzed in percent all the extracts reduce the glucose around 40% after 90 min (Table 1).

### 3.3. Experiments 2 and 3: Hypoglycemic Effects in the Maltose and Sucrose Tolerance Tests

The results of experiment 2 presented in Table 2 showed that, under a maltose curve, the normal control and hyperglycemic groups exhibited a sustained glucose peak from 30 min to 90 min. In this short 90 min timeframe, the glucose values did not return to the normal values (which normally takes 120 min), but the values in the hyperglycemic group were high compared to those in the control group. The control drug acarbose inhibited the glucose peak in a statistically significant way (*p* < 0.05), starting at 30 min. The* Hamelia* and* Neurolaena* extracts did not inhibit the peak or produce a statistically significant hypoglycemic effect (*p* < 0.05), whereas the* Croton* and* Solanum* extracts did not inhibit the peak in a statistically significant way but produced a hypoglycemic effect at 90 min. However, when the data is analyzed in percent the four extracts produce a hypoglycemic effect (around 40%) at 90 min when compared with the hyperglycemic control group.

Under the sucrose curve (Table 3), the results for the normal, hyperglycemic, and acarbose groups were the same as those previously discussed. However, in this case,* Croton* and* Hamelia* produced a statistically significant hypoglycemic effect from 30 min until 90 min,* Solanum* produced an effect at 60 min, and* Neurolaena* only produced an effect at 90 min. Also, when the data is analyzed in percent the four extracts produce a hypoglycemic effect (around 30%) at 90 min when compared with the hyperglycemic control.

### 3.4. In Vitro Test

The results of the enzymatic inhibition of the alpha-glucosidases in vitro are presented in Tables 4 and 5. Acarbose inhibited enzymes from both* Saccharomyces* and rat intestine, whereas the plant extracts inhibited at least 50% of the* Saccharomyces* enzymes, but not the rat intestine enzymes.

## 4. Discussion

In agreement with the traditional use of the plants, all extracts produced a hypoglycemic effect in the first experiment without a sugar load; this effect was like the control drug glibenclamide. When the sugar load was maltose (homogeneous substrate), only* Croton* and* Solanum* produced a hypoglycemic effect compared to the control drug. However, when the sugar was sucrose (heterogeneous substrate),* Croton* and* Hamelia* produced a hypoglycemic effect compared to the control group starting at 30 min, whereas the effects of* Solanum* and* Neurolaena* were observed at 60 min and 90 min, respectively. All extracts inhibited the* Saccharomyces* enzymes but not the rat enzymes.* Croton* produces a hypoglycemic effect without a sugar load as well as with a load of maltose or sucrose; this result suggests that the best effect is produced by* Croton*, followed by* Solanum*,* Hamelia*, and* Neurolaena*.

For* Hamelia* a recent study reports the activity of the compounds catechin, quercetin, epicatechin, and chlorogenic acid over type 1 alpha-glucosidases [11]; the authors conclude that the last two compounds contribute to the antihyperglycemic activity of the plant; the results presented here support the hypoglycemic effect of the plant; also we found activity against type 1 enzymes but we did not find activity against type 2 enzymes. For* Solanum* the alkaloids N-trans-p-coumaroyloctopamine, N-trans-p-feruloyloctopamine, N-trans-p-coumaroyltyramine, and N-trans-p-feruloyltyramine were isolated and the activity correlated with the inhibition of *α*-glucosidases type 1 [12]; our results support this finding but we did not find activity against type 2 enzymes. For* Neurolaena* the sesquiterpene lactones lobatin B and neurolenin B have been isolated and their antiproliferative and anti-inflammatory activities positively tested [17, 25–27]. The phytochemical composition of* Croton* is not reported in the literature. It is possible that the previously reported compounds are responsible for the hypoglycemic activity.

An explanation about why the extracts inhibit only* Saccharomyces* enzymes is the following: Kimura [28] proposed that yeast and mammalian *α*-glucosidases belonged to two different families that differed in their amino acid sequences and their abilities to act on different substrates. The yeast and insect enzymes belong to family I (GH13) and have greater affinity for heterogeneous substrates, such as sucrose or 4-PNGP, whereas *α*-glucosidases from mammals belong to family II (GH31) and have greater affinity for homogeneous substrates, such as maltose. Furthermore, alpha-glucosidase hydrolyzes terminal nonreducing (1→4)-linked alpha-glucose residues to release a single alpha-glucose molecule, such as the molecule present in maltose. In this sense, inhibition of the* Saccharomyces* enzymes by the extracts is more related to an ecological role that enables the plants to defend themselves against insect herbivorism or fungal attacks by inhibiting type 1 enzymes. This finding is important, because it is a common mistake in the international literature for authors to claim a hypoglycemic effect by testing plant extracts on type 1 enzymes combined with a sucrose tolerance test (heterogeneous substrate). Herewith, we confirm that inhibition of the type 2 enzymes is better represented in vivo using a maltose tolerance test.

The results presented here provided evidence based on the use of the plants as hypoglycemic agents, but the effect is not related to inhibition of the type 2* alpha-glucosidase* family. Nevertheless, based on the hypoglycemic effect the use of these infusions could benefit the people suffering from type 2 diabetes.

The four tested plants exert a hypoglycemic effect; in the curve without a sugar load the effect is significant at 30 min while under the sucrose and maltose test the effect is significant after 90 min. In agreement with our results the plants do not exert an antihyperglycemic effect and the hypoglycemic effect can be produced by the stimulation of insulin release; this supposition is based on the short time to produce the hypoglycemic effect in experiment 1 and the fact that the effect is similar to that produced by glibenclamide; this stimulation can be at the gut level through an incretin effect, through direct stimulation of the beta cell, or through the inhibition of the dipeptidyl peptidase-4, but these possible mechanisms of action need further confirmation.

Of the four tested plants,* Croton* exert an effect both under sugars' tests and under a normal tolerance test; these results suggest the potential use of this plant to treat type 2 diabetes; further studies are needed to understand the exact mechanism of action.

## Figures and Tables

**Table 1:** Blood glucose levels under a normal curve.

	Glucose levels in the normal curve [mg/dl]			
Group/time (min.)	T0	T30	T60	T90

Normal control	108 ± 3^b^ 100%	116 ± 4^b^ 107%	114 ± 4^b^ 106%	102 ± 2^b^ 94%

Hyperglycemic control	192 ± 6 100%	184 ± 5 96%	179 ± 4 93%	187 ± 6 97%

Hyperglycemic + glibenclamide 5 mg/kg.	189 ± 4 100%	117 ± 4^a,b^ 62%	105 ± 3^a,b^ 56%	106 ± 4^a,b^ 56%

*Croton guatemalensis *W 20 mg/kg	176 ± 4 100%	163 ± 3^a,b^ 93%	150 ± 5^a,b^ 85%	142 ± 2^a,b^ 81%

*Croton guatemalensis E*W 30 mg/kg	181 ± 4 100%	157 ± 10^a,b^ 87%	123 ± 9^a,b^ 68%	105 ± 5^a,b^ 58%

*Hamelia patens *W 36 mg/kg	201 ± 6 100%	172 ± 5^a,b^ 86%	156 ± 5^a,b^ 78%	143 ± 5^a,b^ 71%

*Hamelia patens *EW 35 mg/kg	173 ± 1 100%	162 ± 5^a,b^ 94%	141 ± 4^a,b^ 82%	141 ± 3^a,b^ 82%

*Neurolaena lobata *W 40 mg/ Kg	191 ± 3 100%	152 ± 56^a,b^ 80%	132 ± 5^a,b^ 69%	139 ± 4^a,b^ 73%

*Neurolaena lobata *EW 57 mg/ Kg	186 ± 3 100%	174 ± 4 94%	156 ± 4^a,b^ 84%	153 ± 5^a,b^ 82%

*Solanum americanum *W *62 mg/kg*	191 ± 5 100%	160 ± 6^a,b^ 84%	138 ± 3^a,b^ 72%	139 ± 3^a,b^ 73%

*Solanum americanum *EW *77 mg/kg*	174 ± 2 100%	163 ± 3^a,b^ 94%	134 ± 3^a,b^ 77%	127 ± 2^a,b^ 73%

The values represent the mean ± SEM; in the same row: a indicates statistically significant differences compared with time 0; in the same column: b indicates statistically significant differences compared with the diabetic control group; *p* < 0.05, n=6.

**Table 2:** Blood glucose levels under a maltose curve.

	Glucose levels in the maltose curve [mg/dl]			
Group/time (min.)	T0	T30	T60	T90

Normal control	119 ± 1 100%	173 ± 6^a,b^ 145%	160 ± 4^a,b^ 134%	154 ± 3^a,b^ 129%

Hyperglycemic control	172 ± 1 100%	293 ± 14^a^ 170%	302 ± 14^a^ 176%	277± 15^a^ 161%

Hyperglycemic + acarbose 3 mg/kg	179 ± 4 100%	238 ± 7^a,b^ 133%	231 ± 8^a,b^ 129%	223 ± 6^a,b^ 125%

*Croton guatemalensis* W 30 mg/kg	173 ± 4 100%	283 ± 21^a^ 164%	279 ± 23^a^ 161%	215 ± 8^a,b^ 124%

*Hamelia patens *W 36 mg/kg	180 ± 5 100%	287 ± 20^a^ 159%	294 ± 18^a^ 163%	257 ± 12^a^ 143%

*Solanum americanum* W 62 mg/kg	182 ± 8 100%	307 ± 18^a^ 169%	289 ± 24^a^ 159%	217 ± 15^a,b^ 119%

*Neurolaena lobata* W 40 mg/kg	172 ± 7 100%	283 ± 23^a^ 165%	310 ± 21^a^ 180%	286 ± 30^a^ 166%

The values represent the mean ± SEM. In the same row: a indicates statistically significant differences compared with time 0. In the same column: b indicates statistically significant differences compared with the diabetic control group; *p* < 0.05, n=6.

**Table 3:** Blood glucose levels under a sucrose curve.

	Glucose levels in the sucrose curve [mg/dl]			
Group/time (min.)	T0	T30	T60	T90

Normal control	114 ± 4 100%	157 ± 2^a,b^ 138%	159 ± 5^a,b^ 139%	148 ± 5^a,b^ 130%

Hyperglycemic control	179 ± 4 100%	270 ± 21^a^ 151%	252 ± 29^a^ 141%	238 ± 27^a^ 133%

Hyperglycemic + acarbose 3 mg/kg	182 ± 7 100%	190 ± 8^b^ 104%	173 ± 6^b^ 95%	178 ± 6^b^ 98%

*Croton guatemalensis* W 30 mg/kg	180 ± 5 100%	215 ± 13^a,b^ 119%	189 ± 4^b^ 105%	165 ± 5^b^ 92%

*Hamelia patens *W 36 mg/kg	171 ± 2 100%	198 ± 5^a,b^ 116%	185 ± 10^b^ 108%	169 ± 3^b^ 99%

*Solanum americanum* W 62 mg/kg	174 ± 4 100%	225 ± 13^a^ 129%	182 ± 9^b^ 105%	153 ± 5^b^ 88%

*Neurolaena lobata* W 40 mg/kg	172 ± 6 100%	225 ± 10^a^ 131%	198 ± 16 115%	161 ± 6^b^ 94%

The values represent the mean ± SEM. In the same row: a indicates statistically significant differences compared with time 0. In the same column: b indicates statistically significant differences compared with the diabetic control group; *p* < 0.05, n=6.

**Table 4:** In vitro results with enzymes from *Saccharomyces cerevisiae*.

Treatment	IC50 *µ*g/ml
Control (acarbose)	105
*Croton guatemalensis*	32
*Hamelia patens*	31
*Solanum americanum*	843
*Neurolaena lobata*	886

**Table 5:** In vitro results with enzymes from rat.

Treatment	IC50 *µ*g/ml
Control (acarbose)	77

## Data Availability

The data used to support the findings of this study are available from the corresponding author upon request.
